# Medical Society of the County of Erie

**Published:** 1907-02

**Authors:** Edward Clark


### SOCIETY PROCEEDINGS.

### Medical Society of the County of Erie.

Reported by EDWARD CLARK, M. D., Acting Secretary,

A MEETING of the Medical Society of the County of Erie was held in the rooms of the Buffalo Society of Natural Sciences, January 14, 1907, at 8:15 p. m., Dr. A. H. Briggs presiding.

In the absence of the regular secretary, Dr. Edward Clark was appointed secretary protem.

Minutes of meeting held October 8th, 1906, also minutes of Council held December 19, 1906, were read and approved.

Dr. Rochester moved that the society proceed to elect a Second Vice-President.

Dr. Grosvenor was nominated by Dr. Rochester but he declined.

Dr. W. C. Krauss nominated Dr. James S. Smith. As only one nomination was made, Dr. Krauss moved that the secretary cast one ballot for Dr. Smith. Dr. Grosvenor objected, and a ballot was taken with the following result: total ballots cast—8, all for Dr. Smith.

The president read a letter from Dr. W. R. Townsend stating that the Society had the right to send nine (9) delegates to the State Society, and suggested that we elect five (5) for two years and four (4) for one year.

Moved and seconded that the society proceed to elect five (5) delegates for two years and four (4) for one year. Carried.

Moved and seconded that we first elect five (5) delegates for two years. The following were nominated:

Lucien Howe, W. C. Krauss—declined, A. H. Briggs, D. W. C. Greene—declined, H. R. Hopkins—declined, A. G. Bennett, A. L. Benedict, F. E. Fronczak, A. T. Lytle.

Nominations were declared closed and ballots taken with the result that the following were elected delegates for two years:

A. H. Briggs, A. L. Benedict, F. E. Fronczak, Lucien Howe, A. G. Bennett.

Nominations for one year:

G. L. Brown, W. M. Ward, A. T. Lytle, J. D. Macpherson.

Nominations were closed, ballots taken and the following were elected as delegates for one year:

J. D. Macpherson, G. L. Brown, A. T. Lytle, W. M. Ward.

Dr. A. T. Lytle, from committee on membership, reported the following applications for membership:

H. P. Frost, transferred from Seneca County Medical Association, of which society he was in good standing; Franklin W.

Barrows, E. A. Hatch, Guerdon Potter, Louis Hengerer, Thos. J. G. Sheehan, Lawrence Hendee, F. A. Mendlein.

The committee on membership reported having received, from applicants, amounts as follows: H. P. Frost, $4.00; Franklin W. Barrows, $4.00; E. A. Hatch, $5.00; Guerdon Potter, $5.00; Louis Hengerer, $5.00; Thos. J. G. Sheehan, $5.00; Lawrence Hendee, $5.00; F. A. Mendlein, $5.00.

Moved and seconded that the chairman cast the ballot of the society for the persons named, which was unanimously carried, and they were declared duly elected.

The committee on status of members, through Dr. C. A. Wall, reported as follows:

"A number of questions were referred to us.

1st. Status of members of the Association not members of the Society.

Under the articles of agreement presented at a previous regular meeting and adopted January 9, 1906, all members of the Erie County Medical Association not members of the County Society were elected to membership and placed on roll of members by unanimous vote taken on acceptance of the rules of the State Society. They are members in good standing, and no further action is necessary.

2d. Members of Association but also members of the County Society but not in good standing in Society at time of amalgamation. Only eight such, of whom three have paid their dues for 1906, and one is now out of the State. A precedent has been established last year, and we advise that the same be done by all: that their back dues be remitted by action of the Society.

3d. Status of all members of the Society as to dues—

Some names of past members now dead are on our rolls, and many members are in arrears, and some names are on the rolls who have never completed their membership, and your committee did not think it advisable, at the present time, to undertake any revision, but wish to report progress and suggest that the treasurer be empowered to employ a collector to see all who are in arrears, at an expense not to exceed twenty-five percent. from the county dues he may collect."

Moved and seconded that the report be received. Carried.

Dr. Grosvenor moved that we consider the report seriatim—seconded and carried.

Dr. Lytle moved, and it was seconded, that the dues of Dr. Bennett be refunded.

Dr. Howe moved, as an amendment, that the dues of all whose names were read by Dr. Wall as in arrears, be refunded. Carried.

Dr. Wall stated that a very large number of members of the old Erie County Medical Society were in arrears for dues and moved that the treasurer of the society be empowered to collect the same at an expense not to exceed 25 percent. of money collected as county society dues.

Dr. Hopkins stated that as this was a time of "Peace on Earth and Good Will to Men" he moved, as an amendment, that the back dues of all members of the society be remitted up to January 1, 1906. Vote taken and amendment lost.

Vote on original motion, namely—recommendation of committee, as moved by Dr. Wall, was then carried.

Dr. Wall stated that there were about two hundred and fifty members of the County Society in good standing.

Dr. Rochester moved that we do now proceed to elect ten (10) delegates to the Eighth District Branch of the State Society. Carried.

Dr. Krauss moved that a ballot be taken for ten delegates—five for two years and five for one year. Carried.

Nominations as follows for two years:

O'Gorman, Mendlein, Krauss, Schroeter, Callanan, W. H. Thornton, E. E. Koehler, Grosvenor.

Ballots were taken with the following result:

Drs. Krauss, Grosvenor, Mendlein, Schroeter, and Ernest Wende, who were declared duly elected as delegates to the District Branch for a term of two years.

On motion, the following were elected, by ballot, as delegates to the District Branch for a term of one year:

Drs. Callanan, O'Gorman, W. H. Thornton, Taylor and Morrison.

Dr. Hopkins, from the Board of Censors, presented the following report which, on motion, was received and placed on file:

*To the President of the Medical Society of the County of Erie.*

Your Censors would respectfully report that it is with distinct satisfaction that we review briefly some of the more prominent events in the work of the censors during the three years last past. This review is for the purpose of making the fact prominent that unusual success has attended our efforts, in directing the attention of those in authority to some of the more notorious medical criminals of our city. In one year the attention of the Criminal Courts was directed to the practice of one Anton J. Weickers, the abundantly advertised boy wonder, and upon trial the so-called boy wonder was sentenced to and did time in the Erie County Penitentiary. The following year the Criminal Courts took notice of the organization and practice of the Vincure Remedy Company, of its aims, its aspirations and its methods, and after trial its Superintendent, one I. A. Finch, so-called doctor, transferred his work from the field made quite large by advertising to the more limited but harmless one of work in the penitentiary.

In the same year the attention of the Department of Education of the State of New York was directed to the business methods of an unlawful corporation, the Atlantic School of Osteopathy, located at the corner of Main and Riley Streets, Buffalo, a short block from the location of the Vincure Remedy Company, Main and Utica Streets, with the result that the Atlantic School of Osteopathy ceased from its unlawful work, the teaching of manual therapeutics and the graduation of persons with diplomas authorizing them to practice medicine under the guise of practicing osteopathy. The number of fraudulent diplomas issued by this Atlantic School of Osteopathy runs well up into the hundreds and your censors are advised by counsel that any of the holders of these diplomas living in this State are liable to arrest.

It was considered by your censors to be a distinct and laudable achievement, to enlist in behalf of the public health, the protective efforts of the District Attorney's Office, of the Criminal Courts, and of the Department of Education of the State, all in a given year, and there were those of our friends who ventured to declare that the year 1905 would ever stand as the year of high record, that in 1905 the censors' clock had struck twelve, and that no subsequent year could make as favorable a showing. Events, however, did not seem to confirm the prophecy and it is the conviction of the censors that the work of the year 1906 in immediate results and in far and wide-reaching future potentiality far exceeds in importance the work of any previous year.

For some time our attention had been directed to the interest and activity of the American Government in its relation to the use of the mails for fraudulent purposes, and we had noted with extreme satisfaction that our national authorities emphatically declined to be used to promote the interests of the advertising medical criminals, declined to share with the more venal of the public press the responsibility and the profits of these criminals, and that the authorities at Washington could be brought into action in the form of fraud orders against certain medical correspondence, and advertisements in the shape of circulars, letters and newspaper notices, forbidding to such the use of the mails. This attitude of our Government gave encouragement and offered help, as the advertising criminals of our city demanded for their detection and suppression powers greater than those of the District Attorney's Office or of the Criminal Courts, or even of the powerful Department of Education of the State of New York.

The scandal of the advertisements of our criminal abortionists had been growing year by year and had become wellnigh intolerable. Our counsel brought this matter to the attention of those in authority at Washington and invoked their aid. An officer representing the Post Office Department was sent here and assigned to the work. The work of this officer covered a period of several months and your censors were able to give him some assistance. His work was performed with effective originality and thoroughness and resulted in demonstrating that our medical criminal class included a group of advertising abortionists large in number, amply equipped, hungry for their victims, brazen with the courage of their depravity. Abundant and convincing evidence to this effect was taken to Washington and there given the consideration its nature and importance richly deserved, with the result that the Buffalo Express of September 25, 1906, contained the following, probably the most startling and important bit of medical criminal news of many years: "By order of the acting Postmaster General, dated September 8, made upon evidence satisfactory to him that the same are unlawful advertisements giving information where abortifacients and criminal operations will be performed, you are directed to refuse to accept for mailing newspapers or other publications containing the following advertisements: Dr. Turver, alias Prospect Avenue Maternity Hospital, No. 101 Niagara St.; Dr. W. A. Crandall, No. 139 S. Division St.; Dr. Linn, No. 10 Broadway; Dr. Wade, No. 485 Main St., alias Medical Home; Dr. Munroe Manges, alias nurse, Box No. 926, Medical Home, No. 777 Washington St.; Dr. Rowe, No. 60 Niagara St.; Dr. Guy Crandall, No. 9 W. Mohawk St.; Dr. Spencer, No. 14 E. Eagle St.; Dr. Ince, No. 66 W. Chippewa St., and by the same order you are further directed to refuse to deliver and treat as fictitious mail received by your office for the following false, assumed, and fictitious addresses used in connection with the above unlawful businesses, Prospect Avenue Maternity Hospital, No. 101 Niagara St.; Woman's Medical Home, No. 485 Main St.; Dr. C. J. Wade, or Dr. Wade, No. 485 Main St; Nurse, Box No. 926; Medical Home, No. 777 Washington Street. You will advise the newspapers of Buffalo that future issues of their newspapers carrying these advertisements will be refused admittance to the mails. In accordance with the provisions of the Postal Laws and regulations you will close all boxes rented by any of these parties." These orders were signed by R. P. Goodwin, Assistant Attorney General for the Post Office Department.

This was followed in the Buffalo Express of September 28, 1906, by the following: "A second fraud order was received at the local Post Office against two more local concerns and was put in force yesterday. The order from the Attorney General in Washington bars from the mail any newspaper carrying advertisements of the Dr. Hewlin Company, No. 485 Main Street, and of Mrs. Park. The order reads as follows: "By direction of the acting Postmaster General you will refuse to accept for mailing papers or other publications containing the advertisements of Mrs. Park and of the Dr. Hewlin Company. You are further directed to refuse to deliver mail to the fictitious addresses, Dr. Hewlin Company and Dr. Hewlin. You will also vacate any postoffice boxes rented by any of these parties. These instructions are additional to those given recently in connection with the other Buffalo abortion offices. Signed by the Assistant Attorney General, as in the former order.

This wholesale suppression of medical criminals we believe is without parallel in the annals of our state. Everlasting gratitude is due from every decent man and woman of this city to those in authority at Washington for this unspeakable relief, this invaluable succor. Your censors are of the opinion that the present situation is favorable for testing the working of our machinery as prepared by law for revoking license to practise medicine and surgery in this State. The thought that the State of New York should license a criminal abortionist, or that an advertising criminal abortionist should continue to prosecute his despicable trade under cover of a license from the State of New York to practise medicine and surgery, is nauseating. The Department of Education has authority to revoke the license to practise medicine when such recommendation shall be made by the board of medical examiners. Your censors urge this matter upon your attention in the hope that it may receive your most thorough consideration, to the end that such instructions as to you may seem expedient under the circumstances may be given to your next Board of Censors.

With distinct reluctance and regret, with feelings akin to shame and confusion, we invite your attention to a phase of our work, to the experience not of one but of many years, to indications which seem to show that loyalty to principles of truth and morality does not abound in all of the members of our profession and does not even seem to be conspicuous in all the members of this society. Again and again it has been our experience that complaint would reach the censors from a member of our society, from one of the laity, or from the police, and the case would be investigated to the end of establishing the fact that the accused was guilty for instance, of the practice of medicine without a license, the same being a crime of misdemeanor, or of falsely personating another practitioner of a like or a different name the crime of felony, or of attempting criminal abortion, or of performing criminal abortion, or the crime of manslaughter, or the virtual crime of murder, and yet the censors would find that the accused person and his crimes was under the support and protection of members of this society in good standing; that this support and protection would appear in different forms, frequently in the shape of letters vouching for his good moral character, or testimony and argument before the Jury, letters and testimony given without any knowledge of the facts and without the least attempt to learn by inquiry from the censors as to the plain and pertinent facts. The work of the censors again and again has been hindered and obstructed by this disloyal and traitorous interference with the official duty of the officers of your society. For years this needless burden has been suffered in silence. It is now, however, the conviction of the censors that forebearance has ceased to be a virtue, that the time has come for plain speech. Names of the members of the society who have been perniciously active in protecting medical criminals, in opposing the work of the censors are not presented at this time. It would seem that the question could be more intelligently and more efficiently considered in the abstract free from the personalities and possible acrimony almost inevitable if the individual members were mentioned. The censors believe, however, that the time has come for this society to give emphatic expression to its convictions as to the importance of certain fundamental truths: that it is before all else desirable that the profession of medicine should include only persons of good moral character; that the habitual perpetration of crimes or criminal acts is a conclusive demonstration that the perpetrator is not possessed of such moral character; that the responsibility of enforcing the provisions of the Penal Code and of the Public Health Law rests upon every member of this society as also it rests upon the Board of Censors; that this Society has the power and the authority to discipline its members; and that this society here and now pledges its full powers in support of any and all of its officers while lawfully engaged in the performance of official duty.

In conclusion we report with regret the receipt of the resignation of our counsel, John Lord O'Brian. This resignation herewith submitted bearing date of December 31, 1906, severs official relations of many years. During all these years Mr. O'Brian has given of his time and his knowledge of medical law to the censors and other officers of your society without stint or limit. The censors bear willing testimony that Mr. O'Brian's services have been of the utmost importance and value, and that whatever of success in doing their official duty has been achieved is in large measure due to the singular devotion, to the high ideals of the purposes of medical law, and the functions of medical societies, of our counsel John Lord O'Brian.

Recognition should also be made of valuable services courteously given by the police through their efficient officers, Superintendent Regan and Captain Collins. Without the sympathy and assistance of the police, much of our work would have been impossible.

Finally we desire to thank our upright and able District Attorney Abbott and Assistant District Attorney Murphy. Their efforts in behalf of decent living have been constant and invaluable.

Respectfully submitted,

H. R. Hopkins
John H. Grant
Francis E. Fronezak
Irving W. Potter
DeLancey Rochester

Dr. Rochester made the following motion:

"Resolved, that it is the sense of this meeting that no member of this society should give certificate of good moral character or letter of recommendation to any individual practising medicine or intending to practise medicine, excepting after careful investigation of all the circumstances in each individual case."

Motion was adopted.

Dr. Rochester also offered the following motion which was adopted:

"That the society directs the Board of Censors to call the attention of the Medical Examiners of the State of New York to the moral status of W. W. Turver, that Examiners may recommend to the Board of Regents a revocation of his license."

Dr. Hopkins nominated Dr. Ernest Wende for Chairman of Public Health Committee.

Dr. Hopkins nominated, but declined. Nominations closed.

Ballot was taken and Dr. Wende was declared duly elected.

Dr. W. C. Callanan moved that report of Board of Censors be published in the Daily Press.

Dr. Grosvenor moved, as an amendment, that such portions be published as may be thought advisable by the Chairman, Dr. Hopkins. Amendment carried.

Dr. John D. Macpherson, the last president of the society, then read his Annual Address which was omitted last year, for want of time.

Dr. Hopkins moved a vote of thanks to ex-president Macpherson. Carried.

It was moved and carried that the address be published in full in the Buffalo Medical Journal.

Dr. DeWitt C. Greene, Treasurer, submitted the following report which, on motion, was referred to an auditing committee composed of the following, named by the Chair: Drs. Grosvenor, Rochester, and Brown.

Dr. Hopkins wanted to know what we received from the State Society for the dues ($500) which we paid last year. He was told that we received the Journal, the Directory, and malpractice defense.

Dr. Wall asked unanimous consent that the By-laws be suspended so that the Treasurer can send out all bills to members for 1907 for $5.00 instead of $6.00. Carried.

On motion, the Council was instructed to fill all vacancies occurring in list of delegates.

Dr. Hopkins moved that resignation of H. C. Leonhardt be accepted and his dues remitted. Dr. Grosvenor moved to lay it on table. Lost. Original motion of Dr. Hopkins was carried.

Dr. H. H. Bingham sent the following letter to the Society:

"I wish you would stop sending me these communications. I am not a member of the Erie Co., Medical Society, for I resigned long ago, and have not been to one of its meetings in years. I do not consider that there is any power that can force one to belong to an organization that he does not wish to continue a member of; and I do not propose to be forced into membership. So please stop this dunning."

Dr. Hopkins moved that the Bingham matter be referred to the Board of Censors with instructions to collect his dues under the law. Carried.

A letter from Dr. Pohlman read by the treasurer was, on motion, placed on file.

Dr. Grosvenor moved that a vote of thanks be extended to the Board of Censors for their excellent report.

Meeting adjourned.

### ANNUAL REPORT OF COUNSEL.

*To the Medical Society of the County of Erie.*

Gentlemen: In accordance with my custom I herewith present the following detailed report of work performed and services rendered by me as counsel since the filing of my last annual report.

So far as the suppression of medical criminals is concerned, it is safe to say that more has been accomplished during the past year than ever before in the history of the society, and it is believed that the statistics below quoted amply support this assertion. The general effect of this work upon the morality and standards of the community must necessarily be considerable and important.

1. Osteopathy.—During the winter of 1906, your counsel carried on an active correspondence with various people with the object of opposing as strongly as possible the passage of the proposed osteopathic measures at Albany. On the 28th of February a delegation from your society, accompanied by your counsel, appeared at Albany before the Joint Legislative committee in opposition to the then pending measure suggested for the regulation of osteopathy. Both Dr. Hopkins and your counsel addressed the Committee upon the subject. A little later, a formal statement of your position and general criticism of the pending measure was drawn up, printed and forwarded to every member of the state legislature, a large portion of the matter therein embraced being furnished by your counsel. In connection with this work your counsel attended several conferences of those interested and furnished those in authority with a considerable amount of data relating to the subject.

2. Parks Case.—Early in June your Chairman called attention of counsel to an advertisement signed by a Mrs. Parks relating to a so-called maternity home. A brief investigation satisfied us that this was a fake institution run by a pair of medical criminals. After further investigation and several conferences with the District Attorney, the house was raided by the police and the criminal caught red-handed in the act of performing a criminal operation. The man was immediately indicted, his woman partner being allowed by the police to go free. Later on the defendant was permitted to go at large upon $1500.00 bail given by one man. In the judgment of your counsel both the bail and the precautions taken by the District Attorney to investigate the same were inadequate. The couple attempted to resume their nefarious business. A further investigation was made by the society with the idea of further prosecution, but the pair immediately fled the city and have not since been located by the police or District Attorney.

3. Wolinska Case.—At the date of my last annual report this case was incomplete. The woman was brought to trial in July with the result of acquittal. She was a Polish midwife charged with the illegal practice of medicine in that she used a scissors to cut the ligaments in the mouth of a tongue-tied child, with the result that the child died. The case against her was thought to be conclusive, but a considerable number of Polish witnesses were sworn by both sides, with the result above stated.

4. By Laws.—In the month of June your counsel was called upon by several members of your society, including Dr. Stockton and Dr. Hopkins, for a formal opinion as to the legality of the new by-laws of the state society, and upon July 12th a formal, lengthy opinion was rendered dealing with this whole subject. In October your counsel furnished a similar opinion in accordance with your request made through Dr. Gram, the secretary, the latter opinion dealing specifically with the proposed new by-laws of the county society. At the request of Mr. James Taylor Lewis, counsel for the state society, your counsel afterward conferred with that gentleman relating to the subject matter and also attended a conference held at the request of Mr. Lewis with several members of your society.

5. Post Office Cases.—Early in the summer your Chairman, Counsel and the Post Office Department reached an understanding for a joint prosecution of all medical criminals in this vicinity who made use of the mails. The work was carried on for a period of time covering several months and involved considerable labor, counsel and advice. As a result of the joint effort so made the Post Office Department early in September, 1906, issued an order denying the use of the mails to the following persons and institutions:

Dr. Rowe,
Dr. Hugh Linn,
Nurse, (the advertising alias of Dr. Monroe Manges)
Dr. Monroe Manges,
Medical Home, (run by a woman named Pyburn for the convenience of several irregular practitioners),

Dr. Cynthia J. Wade, (dead, but whose diploma was in Stevens's hands),

The Hewlin Medical Company,

Mrs. Parks Maternity Home,

Dr. Guy Crandall,

Dr. Ince,

Dr. Spencer,

Dr. W. A. Crandall,

Dr. Turver, alias Prospect Avenue Maternity Hospital,

Dr. Hewlin,

Doctor Hewlin Company,

As a result of this order the parties involved were at once denied the use of the mails and so far as your counsel is aware, none of them has since recovered his right, except Dr. Rowe, who was aided by several prominent local physicians. It is impossible to overstate the effect of this stroke upon the irregular practitioners of the community. Practically all who had been under suspicion and who were not mentioned by name in the fraud order gave up the work. Dr. E. D. Stevens, for instance, who was connected with various irregular institutions, abandoned them and it is understood that he intends to leave the city. The thanks of the society are due to Mr. H. K. Cochrane, the very efficient Inspector of the Post Office Department, whose remarkable diligence and skill made this work successful.

In justice to your officers who did this difficult work, it should be stated that in no case mentioned was the criminal punished by this order on the evidence of less than two witnesses. Practically all of those mentioned were implicated in agreeing to perform abortions. In addition to this, some of the physicians who advertised urinary analysis were charged by the Post Office Department with having furnished false and absurd analyses, finding traces of venereal disease in cold tea and in crystal water.

It is hoped that this wholesale exposure will make less prevalent for a time at least, this horrible business of deliberately slaying by the wholesale unborn children. In this connection your counsel respectfully calls your attention to the fact that a number of members of your society, notwithstanding this fraud order, have written letters of good moral character for several of the irregular practitioners included in the fraud order. They have done this without thoroughly investigating the facts involved and without any conference with or notice to those of your officers who have been engaged in the work of suppressing crime, and their action has been a source of embarrassment to us. It is respectfully submitted that this practice should be stopped. The question is not one of courtesy, but of principle and decency.

6. Turver Case.—For several years your Board has been receiving complaints against Dr. W. W. Turver, but no evidence was ever furnished strong enough to warrant prosecution until the time that the above mentioned drag net investigation was instituted. In the course of that investigation evidence came into the hands of your counsel and Mr. Cochrane which was at once laid before the District Attorney and the police officials, with the result that after a somewhat sensational series of events, Turver was indicted and charged with criminal operation upon one Gertrude Knight. A trial afterwards had resulted in a disagreement by the jury. It is expected that the case will soon again be brought to trial.[1]

7. Mullen Case.—In the month of September the Health Department referred to us the case of a Mrs. Minnie Mullen, charged with having performed criminal operation on a young woman who died shortly thereafter. The woman was at once arrested and indicted for manslaughter. After her arrest a second case was reported to your board and under its direction a careful investigation of the facts was made. In the second case also the patient died. Mrs. Mullen was at once re-arrested and again indicted for the same crime. At present she is at large on bail awaiting trial. Your counsel believes that in her case the evidence of guilt is strong and conclusive.

In addition to the foregoing cases, your board has made a further investigation involving about a dozen persons and institutions suspected of irregular practice. These investigations have not been entirely completed; your officers are still at work upon them and it would be inadvisable to give the particulars thereof at this time, but it may be reasonably stated that further results and prosecutions will follow the conclusion of this work.

During the year many less important questions have arisen and have been attended to by your board and counsel, and some investigations of a minor character have been made. In some of these cases the matters complained of did not require prosecution; in other cases the evidence obtained was insufficient. There have been few days during the entire year when some work has not been done by members of your board and counsel.

In conclusion your counsel respectfully calls attention to the recommendation which he made last year, namely, that some immediate steps should be taken either by means of state legislation, or local ordinance to restrict and regulate so-called medical institutes which are in the main organizations operated by fakirs conducting irregular and irresponsible business, and by their advertisements and practices doing great harm to the welfare of the community. Your counsel desires to express to the members of the society and in particular to the Board of Censors his thanks for their general attitude in regard to the counsel and advice given by him during the past year.

Respectfully submitted,
John Lord O'Brian.

Dated, Buffalo, N. Y.,
December 31, 1906.

1. A second trial resulted in conviction.

